# An Information-Theoretic Approach to Detect the Associations of GPS-Tracked Heifers in Pasture

**DOI:** 10.3390/s21227585

**Published:** 2021-11-15

**Authors:** Cornelia Meckbach, Sabrina Elsholz, Caroline Siede, Imke Traulsen

**Affiliations:** 1 Department of Animal Sciences, Livestock Systems, University of Göttingen, 37077 Göttingen, Germany; Sabrina.elsholz@uni-goettingen.de (S.E.); caroline.siede@uni-goettingen.de (C.S.); imke.traulsen@uni-goettingen.de (I.T.); 2 Campus Institute Data Science, 37077 Göttingen, Germany; 3 Department of Crop Sciences, Grassland Science, University of Göttingen, 37077 Göttingen, Germany

**Keywords:** social networks, pointwise mutual information, association measure, information theory, sensor-tracked animals

## Abstract

Sensor technologies, such as the Global Navigation Satellite System (GNSS), produce huge amounts of data by tracking animal locations with high temporal resolution. Due to this high resolution, all animals show at least some co-occurrences, and the pure presence or absence of co-occurrences is not satisfactory for social network construction. Further, tracked animal contacts contain noise due to measurement errors or random co-occurrences. To identify significant associations, null models are commonly used, but the determination of an appropriate null model for GNSS data by maintaining the autocorrelation of tracks is challenging, and the construction is time and memory consuming. Bioinformaticians encounter phylogenetic background and random noise on sequencing data. They estimate this noise directly on the data by using the average product correction procedure, a method applied to information-theoretic measures. Using Global Positioning System (GPS) data of heifers in a pasture, we performed a proof of concept that this approach can be transferred to animal science for social network construction. The approach outputs stable results for up to 30% missing data points, and the predicted associations were in line with those of the null models. The effect of different distance thresholds for contact definition was marginal, but animal activity strongly affected the network structure.

## 1. Introduction

The importance of understanding and decoding the social structures of livestock becomes increasingly important in modern farming [1,2], since the knowledge can help to make species-appropriate management decisions [2,3,4,5]. Social network analysis has been performed to establish the structures of agonistic behavior in pig groups [6,7,8,9], to estimate the spread of diseases in cattle [10,11,12], and to determine the social structures or agonistic behavior of cattle [10,13,14,15,16,17].

Currently, the variety of methods for observing social or agonistic interactions/contacts is increasing enormously. While in previous times, information about social contacts had to be observed manually by humans and thus restricted by space and time, today’s technology allows the tracking of animals and their contacts in a nearly continuous manner. Video, Global Navigation Satellite Systems (GNSSs), such as the Global Positioning System (GPS), or a wireless local positioning system can be used to track animals independent of time and place [18]. Spacial proximity sensors can be used to monitor close contacts of animals [17], whereas new or future algorithms based on artificial intelligence might be even able to identify social interactions such as grooming or agonistic behavior based on video/image data [19].

However, especially for position data such as GNSS, the identification of real interactions is a problem, and associations of animals have to be estimated based on their pairwise distances [1,4]. Often, distance thresholds are determined beyond which two animals are defined to have a contact [1,13,14,15,17]. The definition of this distance threshold is not trivial [1], and by using a large distance, there is a high chance that for all potential pairs of animals, at least some contacts are observed. Limiting this maximum distance beyond which the two animals are regarded as interacting reduces the number of contacts, and more frequent and close contacts/associations might become clearer. However, the minimal distance an animal keeps between itself and members of the same kind, its individual distance or personal space [20,21,22], as well as the amount of social contacts differ individually. Defining contacts solely on a strict distance level emphasizes those animals with a small social distance and might omit associations among individuals preferring larger social distances. Consequently, the counted contacts contain a large amount of false positive/false negative events by using a too small or large distance threshold. These false positive or negative contacts might be regarded as a kind of noise that needs to be eliminated for plausible predictions of animal associations.

In order to overcome these obstacles, the usage of a variety of association measures in combination with different kinds of background models for the proper determination of significant pairwise associations between animals has been applied [16]. As association measures, we have, for example, the simple ratio index, half-weight index, or square root index [23] to set the number of contacts in relation to the total amount of observations. To focus only on the strongest associations, thresholds were set on these measures beyond which the corresponding dyad is eliminated from the resulting social network [1,13,14,15,17]. With the aim to circumvent the arbitrarily set thresholds, the usage of all associations [24] or the utilization of null models to determine significant associations is common [16,24]. However, the results strongly depend on the chosen null model (also known as the background model), a procedure that generates datasets (i.e., by randomization), which in turn, can be used to compare the original data to [25]. Although for some applications, strategies exist to define a proper null model [4,25,26,27], for other problems (newer problems such as GNSS data), determining the most suitable null model remains challenging [26]. Especially for GNSS data, null model construction by randomizing the raw coordinate data is difficult due to the fact that the autocorrelation of the individual tracks has to be considered and the walking paths of animals should not be interrupted [25]. Further, the construction of huge amounts of background data and related networks can be time and space consuming, increasing dramatically with the data resolution, time period of recording, and number of animals.

In molecular biology, the identification of structurally or functionally important protein sites based on sequence information is a challenging task. During the evolution of protein families, alterations of important protein sites are compensated by alterations of other sites, leading to coupled protein positions. These coupled positions, in turn, can be identified based on their co-evolving behavior; however, phylogenetic background and random noise make it difficult to separate protein sites of coupled mutations from randomly altering positions [28]. Targeting this problem, bioinformatic researchers have established the average product correction (APC) for background noise estimation directly on the underlying dataset without any further background model construction for the determination of important protein sites [28]. Originally, the APC procedure was based on the mutual information of protein sites, but has been adopted in another project for the identification of potentially cooperating transcription factors by using pointwise mutual information (PMI) [29]. PMI is an information-theoretic measure that originated in the field of linguistics for the detection of word associations [30,31,32] and is strongly related to the commonly used association measure square root index utilized in social network construction.

To close the loop, today’s sensor technologies enable the automatic tracking of animals and, thus, their social preferences. However, these data are noisy due to measurement errors and the random co-occurrences of animals. The APC procedure is used to eliminate the random noise or phylogenetic background of sequence-based data. Applying this measure to social network construction would enable researchers to estimate the background directly on the dataset by avoiding the construction of arbitrary null models and maintain in an acceptable range of memory and time consumption. Since the APC procedure was designed for information-theoretic measures and the PMI is suited as an association measure for animal pairings by incorporating their individual preferences, the aim of our study was to combine the PMI and APC for the construction of social networks based on the sensor-tracked positioning data of animals.

In order to perform a proof of concept that the combination of both strategies can be adopted in social network construction, we investigated one month of GPS data of grazing heifers and conducted an association study followed by the social network construction of heifers in a pasture.

## 2. Materials and Methods

All animal treatments were in accordance with EU legislation (Council Directive 86/609/EEC). The sensor technology used was noninvasive and commercially available.

### 2.1. Animal Housing and Sensor Technology

The study took place in autumn 2019 for 29 days (24 September 2019 00:00 a.m.–22 October 2019 11:59 p.m.) in Lower Saxony, Germany. Eight pregnant heifers (see Table 1) aged between 22 and 25 months were equipped with GPS collars and activity sensors. The heifers were kept on intensively managed pastures together with two other cattle not equipped with sensor technology. The considered pasture had a size of 5 ha and was divided into an eastern (2.4 ha) and a western (2.6 ha) part. At the study start, the eastern part was newly opened and freely accessible to the animals. Before, they spent already 12 days on the western pasture part, fully equipped with sensors for adaptation.

The activity data were tracked using the commercially available activity sensor *Ice Tag*, (IceRobotics Ltd., https://www.icerobotics.com (accessed on 5 November 2021)), which were designed for research. The device contains a 3-axis accelerometer and has a weight of 130 g [33]. The sampling rate is 16 Hz [33], and we used a 1 min resolution to store animal activity. The sensor was attached to the cows’ rear leg and was used to determine for each sample point the proportion between the cows standing or lying during one time interval (1 min). The activity data were retrieved at the end of the grazing period by using the software IceManager 2014 (Version 3.0.0.1).

As the GPS sensors, we used the commercially available *Vertex Plus Collar* (battery type 2D, Vectronic Aerospace GmbH, https://www.vectronic-aerospace.com/vertex-plus-collar/ (accessed on 5 November 2021)) for the positioning data. The device stores the Coordinated Universal Time (UTC) date and time, GPS coordinates (longitude, latitude, height), the number of satellites, and the dilution of precision (DOP) for each sample point and has a total weight of about 1280 g (collar and battery). We used a one-minute resolution for position tracking and received the data by using the GPS Plus X software (Version 10.3.1) at the end of the grazing period. The accuracy of the used device was stated by the manufacturer to be within 8 m and 15 m as the mean, but should be far more accurate under proper conditions. In order to determine the quality of our GPS records of the heifers, we selected all lying events of each animal that lasted more than 60 min. Since the animal was not walking, the variance of the records should be relatively stable. However, it has to be claimed that head shaking or head movement might be contained in these events. For each lying event, we calculated the mean coordinates (using the *geomean* function of the *geosphere* package) and calculated the standard deviation for each direction (longitude and latitude) in meters. The standard deviation in the latitude direction was 1.04 m and 0.8 m for the longitude direction. Afterwards, we calculated the circular error probability (CEP) (50% radius), which was 1.10 m, and twice-distance root mean square (2DRMS) (95% radius), which was equal to 2.66 m.

Tracking the animals with a one-minute resolution, we ended up with about 41,760 data points per animal in total (the exact numbers are given in Table 1). Regarding the GPS quality, on average, 6.2 ± 3.2 satellites were used for position estimation, and the average DOP was 1.5 ± 0.3 (see Table 1 for the data of individual sensors).

### 2.2. Social Network Construction

**Figure 1:**
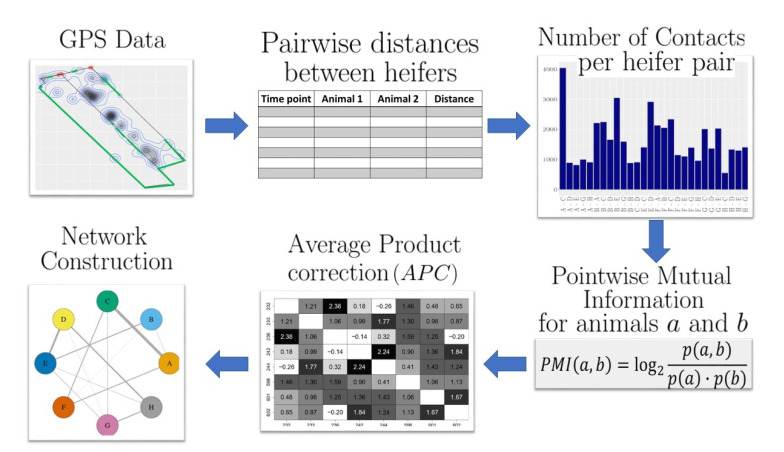
Workflow of social network construction. Based on the GPS data, the pairwise distances at each tracked time point were calculated for all heifer pairs. Based on these distances, all pairwise contacts were counted by using a predefined distance threshold. These numbers of contacts in turn were used to calculate the (positive) *pointwise mutual information* (PMI), and background co-occurrences of heifers were eliminated by using the average product correction (APC) procedure. Finally, the social network was constructed where edges refer to associations between the heifers, i.e., the corresponding PMI values were >0 after the APC procedure.

The overall workflow of the proposed method is shown in Figure 1 and explained in detail in the following.

Data processing, network construction, and graphic generation were conducted with R [34] (Version 3.6.3) by using the following packages: *dplyr* [35] (1.0.7), *extrafont* [36] (0.17), *geosphere* [37] (1.5.10), *ggplot2* [38] (3.3.5), *igraph* [39] (1.2.6), *NetworkDistance* [40] (0.3.4), *plot.matrix* [41] (1.6), *ggnewscale* [42] (0.4.5), *sjmisc* [43] (2.8.7), *stringr* [44] (1.4.0), and *lubridate* [45] (1.7.10).

#### 2.2.1. Pairwise Distances between Heifers

In the first step, the GPS data with one-minute resolution were used to determine the distance between each two animals for each time point. For this, we used the Haversine distance (R package *geosphere*) to access the distances between GPS coordinates in meters. This resulted in a list of pairwise distances for each heifer pair and each time point.

#### 2.2.2. Contacts between Heifers

Based on these distances, we counted the number of contacts between two animals by setting a maximal distance threshold beyond which two animals were defined to have a contact. Thus, we considered only those contacts $c_{a,b}$ where the distance $d_{a,b}$ between two animals *a* and *b* did not exceed a predefined threshold *d*. Afterwards, we counted for each pair of heifers the number of contacts.

#### 2.2.3. Pointwise Mutual Information

In order to measure the level of association (positive relationship) between the animals, we calculated the PMI between each pair of heifers. The PMI measures the probability of coincident occurrence of two variables (i.e., animals) with respect to the probability of independent occurrence [31]. The PMI(a,b) [29,31,46] for two heifers *a* and *b* is calculated as:

(1) $$PMI\left(a,b\right)=log_{2}\frac{p(a,b)}{p(a)\times p(b)}$$

The joint and marginal probabilities for the occurrence and co-occurrence of the heifers are defined as follows:

(2) $$p\left(a,b\right)=\frac{f(a,b)}{n}$$

(3) $$p\left(a\right)=\frac{f(a)}{2n}$$

where f(a,b) is the number of time points heifers *a* and *b* have a contact and *n* is the number of total contacts between animals. f(a) is the number of times heifer *a* is involved in a contact. Defining the probabilities in that way, we ensured that $\sum_{a,b}p\left(a,b\right)=1$ and $\sum_{a}p\left(a\right)=1$.

Afterwards, we transferred the PMI to the *positive pointwise mutual information* (PPMI) [46], since the following steps were restricted to positive values. The PPMI matched the positive PMI values and was set to 0 if the corresponding PMI value was negative [46].

(4) $$PPMI\left(a,b\right)=\left\{\begin{array}{cc} PMI(a,b), & if\;PMI(a,b)>0\\ 0, & otherwise \end{array}\right.$$

#### 2.2.4. Average Product Correction

In order to increase the reliability of the estimated social associations, we eliminated the background noise to some extent by using the *average product correction* (APC) procedure [28]. The APC(a,b) [28] of two heifers *a* and *b* is defined as:

(5) $$APC\left(a,b\right)=\frac{PPMI\left(a,\bar{x}\right)\times PPMI\left(b,\bar{x}\right)}{\bar{PPMI}}$$

where, for each pair of animals, the background level regarding their PMI values is estimated based on the average PMI values ($PPMI(a,\bar{x})$) the individual animals share with other heifers with respect to the overall average ($\bar{PPMI}$).

(6) $$PPMI\left(a,\bar{x}\right)=\frac{1}{n}\sum_{x\neq a}PPMI\left(a,x\right)$$

(7) $$\bar{PPMI}=\frac{1}{n^{2}}\sum_{x\neq y}\sum_{y\neq x}PPMI\left(x,y\right)$$

Afterwards, the estimated background APC(a,b) was subtracted from the original PPMI(a,b):

(8) $$PPMI^{APC}\left(a,b\right)=PPMI\left(a,b\right)-APC\left(a,b\right).$$

Finally, we defined two animals *a* and *b* to have a social connection/being associated if their corresponding $PPMI^{APC}\left(a,b\right)$ value was positive.

#### 2.2.5. Network Construction

Constructing the social networks, the heifers were represented as nodes in the network, and there was an edge between them if they shared a positive relationship, meaning their $PPMI^{APC}\left(a,b\right)$ value was positive.

For a better understanding and interpretation of the functionality of both PMI and the APC procedure, please see Appendix A.2.

### 2.3. Validation of the Application

#### 2.3.1. Influence Factors of Network Construction

In order to investigate the influence of different distance thresholds *d* for heifer contact definition on the social network structure, we tested thresholds of d= 5 m, 2.5 m, 1.5 m, and 1 m.

The activity sensors determine with a one-minute resolution the relative time the animals spend lying or standing. In order to compare the social networks resulting from different animal activities, we constructed networks based on the data of solely * (i) standing or (ii) lying animals. Only records of animals were taken into account when the animal was entirely (the whole minute of the record interval) standing or lying.

#### 2.3.2. Robustness

A robust method should produce stable results and should therefore not be affected by a certain amount of missing data.

In order to demonstrate the robustness of the approach, we systematically eliminated random recordings of the original GPS dataset and compared the resulting networks with the original one. For this comparison, we used the Hamming distance, which is defined for two undirected networks $g_{1}$ and $g_{2}$ of the same number of nodes *N* by:

(9) $$H_{g1,g2}=\frac{|E_{g1}\cup E_{g2}\left|-\right|E_{g1}\cap E_{g2}|}{N(N-1)/2}$$

where $E_{g1}$ and $E_{g2}$ are the edges of graph g1 and g2, respectively [47]. The Hamming distance focuses on the number of different edges between the two graphs; thereby, different edges can be either exchanged, newly present, or absent. If $H_{g1,g2}=0$, the graphs g1 and g2 are identical.

#### 2.3.3. Comparison with Existing Methods

For a proper evaluation of the detected edges, we performed a common analysis by using a null model to determine significant associations between heifers (as described by [25]). In general, a null model is a procedure that produces datasets (e.g., by simulation or randomization), which, in turn, are used to test the observed data against [25,48]. Since the construction of proper null models based on the raw coordinate data is very challenging [25], we took the pairwise distance table of all time points and shuffled the distance column 1000 times by keeping time and animal pair. Afterwards, we determined significant associations in two ways: (i) contact numbers and (ii) PMI values. The p-values were calculated following [25].

## 3. Results

### 3.1. Investigation of Animal Contacts

**Figure 2:**
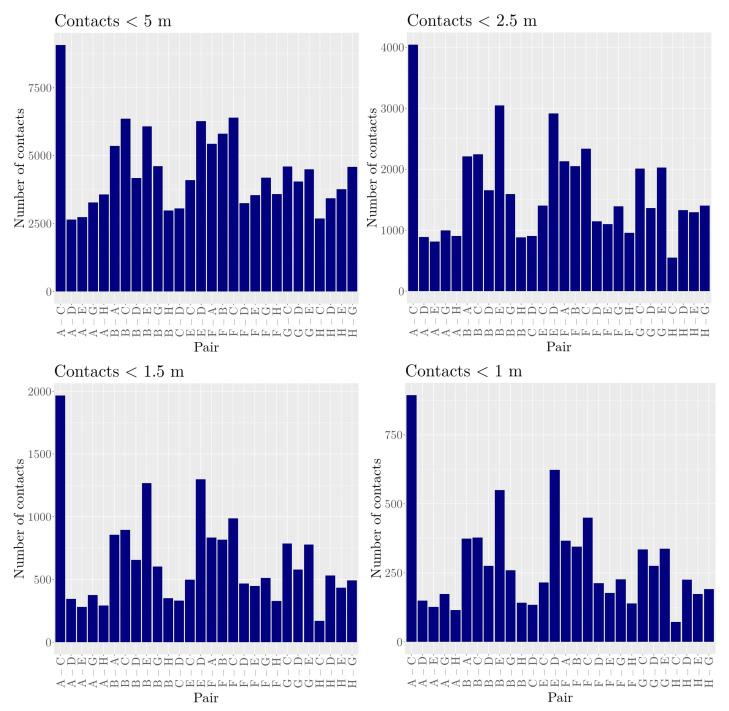
Number of contacts for all dyads. Number of contacts, i.e., time points in minutes, the two animals shared a distance below a threshold of 5 m, 2.5 m, 1.5 m, or 1 m, respectively.

The number of contacts with respect to different distance thresholds (5 m, 2.5 m, 1.5 m, and 1 m) is shown in Figure 2. Despite the overall number of contacts decreasing with increasing distance threshold, the most obvious properties of the distributions remained, i.e., the heifer pair A–C had the largest number of contacts for all distance thresholds. It should be mentioned that the pairs B–C and B–E had nearly the same number of contacts using a distance threshold of 5 m, but the pair B–E showed much more contacts for all other distance thresholds. Applying a $\chi^{2}$-test to determine whether there was a significant difference in the frequencies of contacts between each two distance thresholds, we found that except for the contact distributions 1 m and 1.5 m, all other combinations were significantly different (p<0.05).

**Figure 3:**
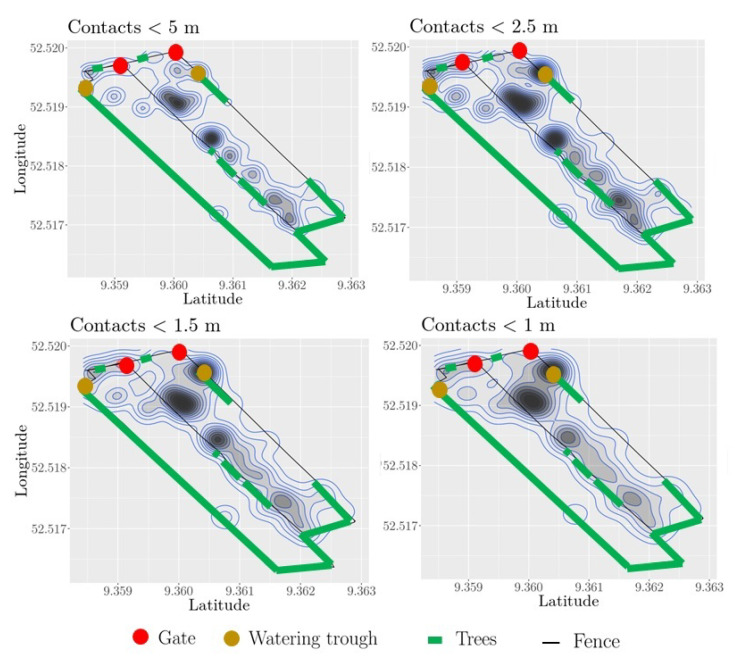
Contact zones of heifers. The dark zones mark the areas of a high number of pairwise contacts, i.e., the distance between heifers is less than a distance threshold (1 m, 1.5 m, 2.5 m, 5 m). Black thin lines indicate the fence of the pasture; the middle fence separates the eastern from the western part. The gate (red) at the middle fence is open, and the heifers can move freely between the eastern and western pasture part.

We further investigated the contact locations in the pasture to indicate to what extent these locations might change in response to different distance thresholds. The contact zones of the animals are depicted in Figure 3 and were spread through out the entire eastern part of the pasture, the newly opened pasture part. A clear contact zone was the eastern watering trough; this contact zone became apparent by decreasing the distance threshold. Other hot spots for contacts were the locations close to the middle fence, which separates the eastern and western pasture parts. The contact zones close to the middle fence were similarly located for the individual distance thresholds with some changes in their intensity that might be related to the different number of general contacts.

**Figure 4:**
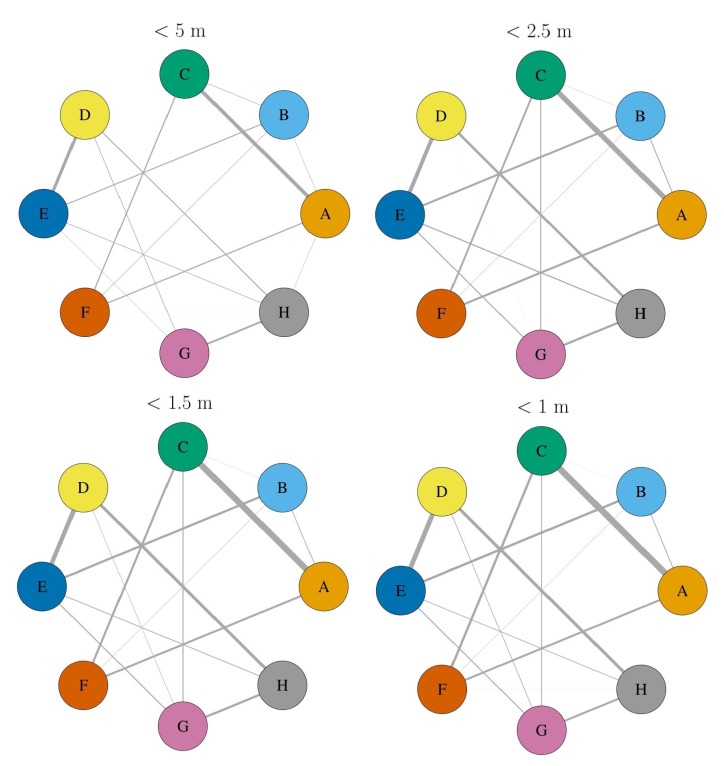
Social networks of the eight heifers in the pasture based on the $PPMI^{APC}$ values for different distance thresholds used to define a contact. Heifers are presented as nodes and labeled by their ID, and edges indicate a positive association according to their $PPMI^{APC}$ values. The edge width reflects the strength of this association. Please note that Heifers H and F are connected in the networks of the 1 m and 5 m distance thresholds, but since this association is close to zero, it is barely visible.

Based on the observed pairwise contacts between heifers for the individual distance constraints, we constructed social networks in the way described above for all distance thresholds (1 m: network N1; 1.5 m: network N1.5; 2.5 m: network N2.5; 5 m: network N5). The networks are shown in Figure 4 and appear to be very similar regarding their general structure. The networks N1.5 and N2.5 consisted of 14 edges, whereas the networks N1 and N5 had 15 edges. The networks N1.5 and N2.5 were identical, and 13 edges were present in both networks. A comparison of the individual networks based on their edges is shown in Table A1.

Regarding the social animal herd structure, we investigated the number of connections per animals, in network theory referred to as the degree of a node. A *hub node* in this context is an outstanding node regarding its degree, i.e., it is much more connected than most of the other nodes. For the underlying social networks of heifers based on different distance thresholds, no hub node could be found. However, Heifers B, E, F, and H all had four associations with other heifers, and four was the largest degree. Heifer C had the highest weighted degree (sum of related $PPMI^{APC}$ values). The strongest connections were formed between Heifers D and E, both Frisian Holstein, same age (nine days difference), and same calving dates (two days difference), as well as between A and C, both crossbreed, identical age (three days difference), and close calving dates (three days difference).

### 3.2. Investigation of Animal Activity

**Figure 5:**
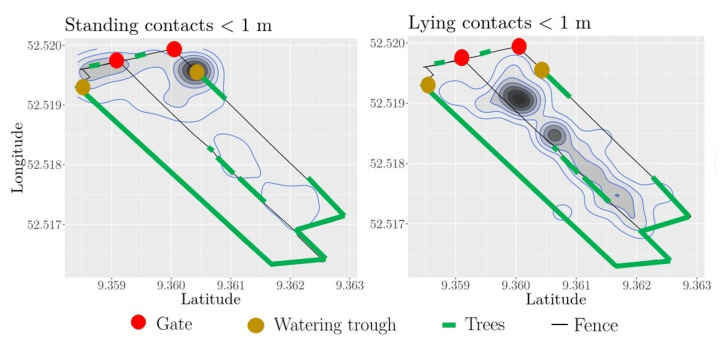
Contact zones of standing and lying heifers, respectively. The dark zones mark the areas of a high number of pairwise contacts, i.e., the distance between heifers is less than the distance threshold of 1 m. The distribution of contacts is shown by blue lines. Black thin lines indicate the fence of the pasture; the middle fence separates the eastern from the western part. The gate (red) at the middle fence is open, and the heifers can move freely between the eastern and western pasture part.

In order to investigate the different outcomes of social network construction based on animal activity, we separated the dataset into (i) both animals lying and (ii) both animals standing. For each of these two cases, we show the contact zones in Figure 5, choosing a distance threshold of 1 m. Contacts between standing animals were observed most frequently at the watering trough, as well as at the gate connecting the two pasture parts. Contacts between lying animals were most frequent in the north close to the middle fence, the area the animals used to lie down.

**Figure 6:**
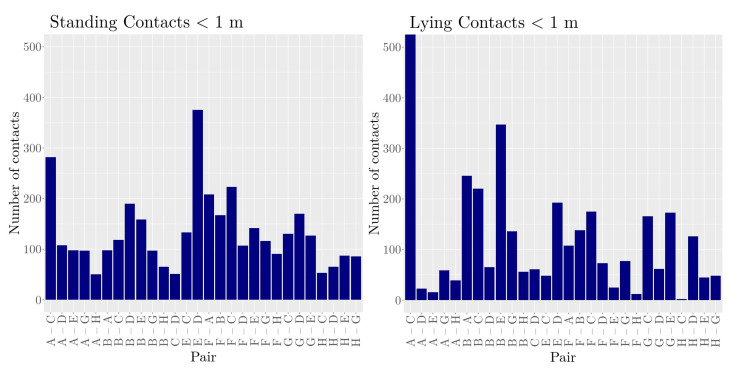
Number of contacts (distance < 1 m) for standing and lying animals, respectively.

Second, we investigated the number of contacts between standing and lying animals. As shown in Figure 6, the general distribution of pairwise contacts differed between standing and lying animals ($\chi^{2}$-test p<0.05). For the lying animals, the differences between the individual pairs were stronger compared to the standing heifers, and also, the top contact pairs differed between standing and lying. For the standing animals, the pair E–D showed the most contacts, whereas for lying, Animals A and C formed the strongest pair. Regarding the general contact frequencies between standing and lying heifers, there was no significant difference (Wilcoxon rank-sum test p>0.05).

**Figure 7:**
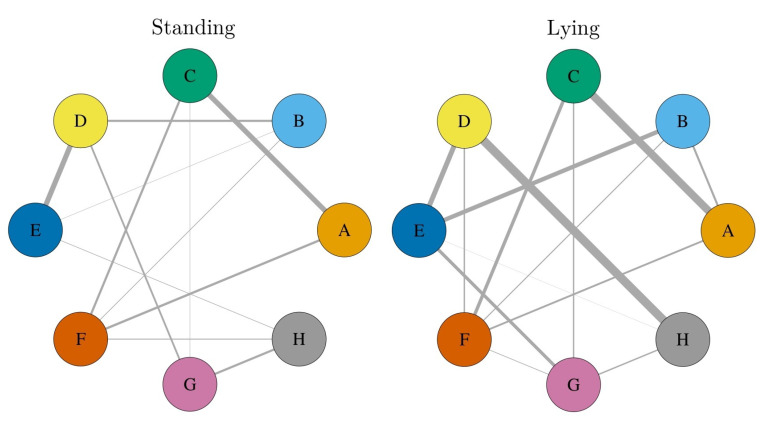
Social networks constructed for standing and lying heifers, respectively. Nodes represent heifers labeled by their ID, and edges represent identified contacts after applying the APC procedure. The edge width reflects the strength of the association.

Based on these numbers of contacts, we constructed social networks for the lying and standing heifers, respectively, by using the described methodology. The networks are shown in Figure 7. Both networks included all eight heifers equipped with GPS tags. The network for standing heifers consisted of 13 social relations and the network of lying heifers of 14. Comparing both networks, nine links were consistent. Thus, for the standing network, four dyads were unique (B–D, D–G, B–H, F–H) and five for the lying network (A–B, D–F, E–G, F–G, D–H). The most striking difference between the networks was the connection between Heifers D and H, which was strongly represented in the lying network and not present in the network of standing heifers. A glimpse back to Figure 6 shows that this pair had the most contacts of lying animals for Heifer H. In contrast, it was even underrepresented among the standing animals.

Investigating the social structure of the heifers, for the lying animals, Heifer F had, with a degree of five, the most predicted associations with other heifers. For the standing heifers, no clear hub node could be identified, but three heifers (B, F, H) had four associations. Regarding the weighted degree (sum of all $PPMI^{APC}$ values of adjacent edges), the node representing Heifer D had the highest-weighted degree in the standing and lying network.

The strongest connection in the standing network was formed between Heifers D and E; both are the Holstein Frisian breed and share the same age and very close calving dates. The second strongest pair was formed by Heifers C and A, both crossbreeds, with a similar age and calving dates. These two dyads also formed the strongest pairs of the lying network. Additionally, in the lying network, the association between Heifers D and H stood out. These two heifers differed in their age by about three month; both are the Holstein Frisian breed, and their calving dates had more than a 100-day difference.

### 3.3. Investigation of the Method’s Robustness

In order to verify the robustness of the presented approach, we randomly removed parts (10% to 60%) of the original GPS input data and constructed networks based on the remaining GPS dataset consisting of 90%, 80%, 70%, 60%, 50%, and 40% of the original data. We compared the resulting networks to the one of the original dataset size by using the Hamming distance. For contact definition, we chose a distance threshold of 1 m for the construction of all networks.

**Figure 8:**
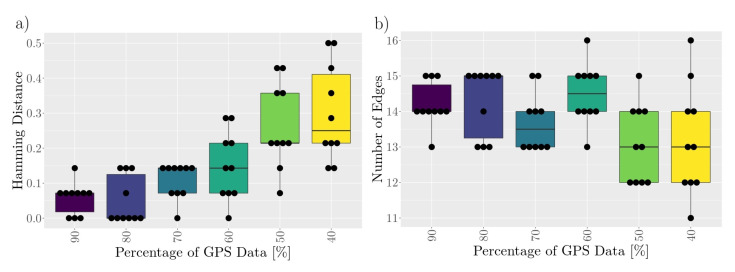
Robustness of the APC procedure applied for social network construction to the loss of GPS data. Randomly, fractions of data were removed in a way that only 90%, 80%, 70%, 60%, 50%, or 40% of the original data remained for network construction. This was done ten times per fraction. Afterwards, the constructed networks based on the incomplete data were compared to that of the original GPS dataset by using (**a**) the Hamming distance and (**b**) the number of edges. The original network consisted of 15 edges. In all networks, the number of nodes remained at eight. A Hamming distance of 0 indicates identical networks; a Hamming distance of 0.5 indicates 7 different edges (exchanged/newly present/absent).

The results are presented in Figure 8. With inclining losses of data, the difference of the original from the networks of lost data increased. Considering networks constructed of 90% or 80% of the original data, the Hamming distance indicated only small differences between the networks and the original one regarding their edges. In fact, a Hamming distance of 0.14 indicated a difference affecting two edges. None of the networks constructed with 50% or 40% of the original dataset were equivalent to the original network. The observed maximum Hamming distance of 0.5 indicated a difference between the networks of seven edges (exchanged, newly present, absent).

The network density, presented here by the number of edges, showed increasing variation with increasing loss of GPS data. The original network had 15 edges; constructing networks by using 90% or 80% of the original data, the number of edges varied between 13 and 15, whereas for networks formed by using only 40% of the GPS data, some networks had 11 or 16 edges.

### 3.4. Validation of APC Application

**Figure 9:**
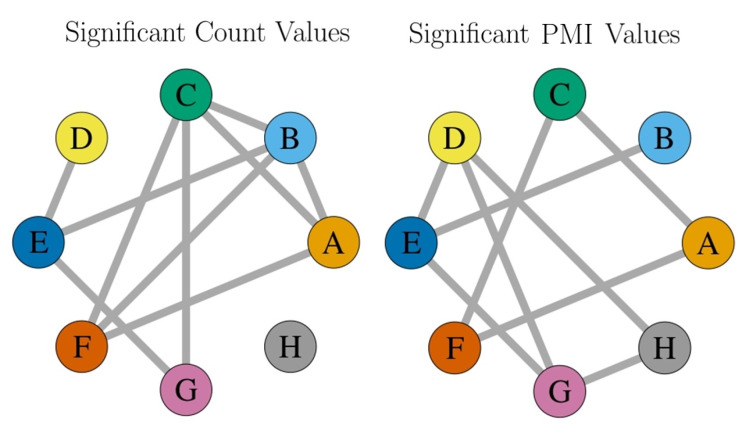
Network constructed from significant count values and significant PMI values in comparison to the null model.

In order to compare the application of PMI combined with the APC procedure to the common method, i.e., the comparison to the null model, we shuffled the pairwise distances of the heifer pairs 1000 times and determined (i) the number of contacts (1 m distance threshold) and (ii) the PMI values for each of the 1000 samples. Afterwards, we determined for each number of contacts and each PMI value of the original data whether it was significantly different from the data generated by the null model. Based on these calculations, we constructed networks of significant contact counts and PMI values, respectively. The resulting networks are presented in Figure 9; since the edges were either significant or not, the width of the edges is uniform.

The comparison between the network produced by the APC procedure and the two networks of (i) significant contact counts and (ii) significant PMI values is given in Table 2. All of the edges contained in the two significant networks were present in the $PPMI^{APC}$ network. Six of them were present in all three networks, and two edges (E–H and F–H) solely occurred in the $PPMI^{APC}$ network.

## 4. Discussion

### 4.1. Investigation of Different Distance Constraints

Since the distance between cattle reflects their social relationship [16,18,49,50,51], commonly, by using point location data (such as GNSS records), a distance threshold is set for the determination of contacts, beyond which two animals are defined to have a contact [1,13,14,15,17]. Increasing or decreasing this distance threshold influences the number of observed contacts, i.e., less contacts are observed for more strict distance thresholds. Thereby, the number of false positive contacts, i.e., the animals pass each other without any intention to be close to each other, drops. On the other hand, strict distance thresholds might lead to an under-representation of animals with a larger individual space. We investigated four different distance thresholds of 1 m, 1.5 m, 2.5 m, and 5 m. Our results showed that although the number of contacts declined by decreasing the threshold, the general distribution of the number of pairwise contacts remained and appeared to be relatively stable. As a consequence, the corresponding social networks were very similar for the different distance thresholds. This finding is in line with a study of Rocha et al., where they investigated the influence of different distance thresholds (1.25 m ± 0.25 m) of pen-housed dairy cattle. They concluded that although the general number of contacts decreased/increased, the resulting networks did not change qualitatively [13]. Transferred to cattle behavior, this aspect might indicate that there is no heifer with a considerably larger individual space than the others, and the general number of false positive contacts dropped equally with the number of contacts, thus not affecting the general distribution.

### 4.2. Investigation of Different Activities

The identification of associations between animals might also be affected by the activity the animals are performing. For example, during grazing, animals are within their herd, and being close by their preferred herd mate might not be as important as lying down for sleeping or ruminating. Shiyomi found that the distances, as well as the distance variations were much bigger during grazing than in the resting phase [52]. Further, associations between cows are not necessarily fixed, but can be context dependent [16,53], and thus, different social networks might be observed from different functional areas [16].

We investigated the number of pairwise contacts for lying and standing animals by using a distance threshold of 1 m. Although the general number of contacts did not differ significantly between the two scenarios, the general contact frequency differed strongly between standing and lying animals, which is in line with the expected behavior of heifers in pasture. Further, the contrast between the animal pairs was much clearer for the lying animals. In consideration of the fact that lying is a stationary behavior and not directly comparable with walking, our findings suggested that heifers have preferred herd mates and lie down next to them. The associations of the standing network were in general weaker compared to the lying network. This was supported by Gygax et al., who found that social networks of dairy cattle become less strongly connected from the lying and feeding area to the activity area [16].

Regarding the constructed social networks for lying and standing, around 2/3 of the edges were consistent, whereas 1/3 differed. Since the contact distribution of the standing animals was not as clear as that of the lying ones, we suggest that the amount of random contacts by passing by affected the analysis, and a differentiation between loose connections to random connections might not be possible. Still, the strong connections presented in the lying and standing networks matched. Following our findings, we would like to point out that the activity the animals were carrying out was of major importance for the construction of social networks and that solely focusing on distances might not be sufficient, especially for identifying loose, but still present associations.

### 4.3. Robustness and Validation

Our results showed that removing up to 30% of the underlying original data affected only up to two edges in the social network. The elimination of more data points led to more diverse networks, and at most, seven edges were different, increasing the chance of misinterpretations. With that, the method appeared relatively stable regarding its general findings, since the number of nodes remained, as well as most of the detected associations.

Regarding the reliability of the detected associations between heifers, we constructed two different null models. The first solely focused on the number of contacts, thus not affected by the association measure used. The second one relied on the PMI, an information-theoretic association measure that preprocessed the data more than the other by incorporating the knowledge about the frequency that the respective heifers participated in other pairings.

Both null models are appropriate to compare with the APC procedure, since all approaches intend to identify the most important edges of the networks. However, it has to be mentioned that Heifer H was not connected to any other heifer in the network of significant contact frequencies. This heifer had in general a low number of contacts, but was unlikely not to be connected to the other heifers of the herd. This finding might point out the requirement to further process the contact numbers (i.e., by PMI) to determine all associations with respect to the individual contact behavior of the animals. Although both networks were developed by a prenetwork permutation-based null model, only about 2/3 of the edges were identical. This underlines the statement that the output networks strongly depend on the chosen null model [25].

All of the edges determined by the null models were present in our network, and therefore, the significance for all edges, except two (E–H and F–H), could be proven. Whereas the association of F–H determined by $PPMI^{APC}$ was close to zero, and thus very poor, the association of E–H was stronger, but still was the second-weakest connection of Heifer H. Whether the presence of these two edges is appropriate might depend on the application aim or research question. For our application, the identification of social associations between heifers, the identification of these edges was not disadvantageous and helped identify the position of heifers in the social herd structure.

### 4.4. GNSS Technology

In our study, we used commercially available GPS collars, including a standard GPS receiver using absolute positioning. Based on lying events, we determined the quality of the used GPS collars for the underlying study conditions. In consideration that there might be movement of the receiver due to animal’s head motion, the accuracy was acceptable, but still in the range of meters (CEP = 1.10 m, 2DRMS = 2.66 m). Thus, several of the tracked contacts might be regarded as noise due to measurement errors, i.e., the ground truth distance between the animals might be 3 m, but we determined a contact using a distance threshold of 1 m. However, since the differences between the different distance thresholds that were above or below the device accuracy, our method seemed to be able to identify the signal in the underlying data.

It has to be mentioned that there exist strategies based on GNSSs that promise to be much more accurate than common GPS sensors. Next to the commonly used GNSS, GPS, other GNSSs exist such as the *European Global Navigation Satellite System* (Galileo), *Global’naya Navigatsionnaya Sputnikova Sistema* (GLONASS), and the *Chinese BeiDou Navigation Satellite System* (BeiDou) [54,55]. The combination of these systems, referred to as multi-GNSS, promises to be beneficial regarding a shorter positioning convergence time [55,56], as well as position accuracy due to the increased number of satellites and the better constellation between satellites and sensors [54,55] after the correction of ambiguities [56]. While solely using multiple GNSSs, the accuracy might stay in the range of meters, utilizing *real-time kinematic* (RTK) positioning, and differential correction can lower the error in the range of centimeters under perfect conditions [57].

Investigating the social behavior of Merino Ewes, Keshavarazi et al. constructed an RTK-corrected multi-GNSS device named RTK rover that was attached to the animals by using a dog harness [57]. The accuracy of the receiver was determined to be 20 cm, and the researchers compared video observations with GNSS records, concluding that the RTK rover correctly determined the position of animals relative to each other [57]. Although the usage of this device appears to be not yet practical for continuous recording of animals for a long time, the authors underwent an important step by investigating new technologies for animal monitoring.

We assumed that, in the near-future, improved multi-GNSS receivers will be commercially available as collars with affordable costs and will allow a much more accurate tracking of animal behavior. Focusing on our proposed methodology, the more accurate predictions will allow a better estimation of the background co-occurrences, i.e., random or not intended co-occurrences of animals, giving more hints to understand the complex social behavior of cattle or any other social animal species equipped with multi-GNSS sensors. Further, the networks might become more accurate, helping to make more precise management decisions.

### 4.5. Association Measures of Animal Relationships

Commonly used association measures are the *half-weight index* or the *simple ratio index*. These measures focus on the number of co-occurrences of animals in relation to the general occurrence of the animals and are powerful regarding manual animal behavior observations. Technologies, such as GNSS, GPS, wireless local positioning systems, or even cameras (recording from a proper perspective by including automatic object detection), promise to be valuable for precision livestock farming [18,58]. All these technologies provide one record per animal and time interval, and consequently, the number of general occurrences of the animals in the dataset is equivalent. With that data, the mentioned association measures are nearly as good as focusing solely on the number of co-occurrences. In turn, the *square root index*, as well as PMI depend on the probabilities of the co-occurrence and single occurrence of the animals. These probabilities can be defined in an appropriate way for the data type. Originally, the probabilities were defined by the number of observations of the animals [23]. We defined the probabilities of an animal pair based on the general frequency of co-occurring animals, i.e., number of contacts of animals. The probabilities for the individual animals were based on the number of animals occurring in pairings. With these probability definitions, we set the probability of the co-occurrence of two heifers in relation to of their individual probability to be found paired. Further, using these definitions, we increased the final PMI values, and thus, the number of pairings exceeding an PMI value greater zero was increased. This was intended since our aim was to further use the APC procedure for the identification of important associations. The APC procedure has been developed for mutual information values that are positive by definition. As an additional comment, the negativity of PMI might also be circumvented by using the PMI formula without the logarithm or the *square root index*. However, the presence or absence of a logarithm can change the general distribution of the values. Further, the APC is not applied to an information-theoretic measure; associations occurring less than expected by chance remain in the data, and the process of filtering might not be predictable. Nonetheless, if the definition of probabilities cannot be adopted in a proper way and leads to an unexpected and unrealistic multitude of negative PMI values, this way of acting can be considered.

### 4.6. Heifer Social Associations

Analyzing the social associations between heifers, all networks (all activities, lying, standing) showed the strong connection between Heifers D–E and A–C, which fit perfectly regarding breed, age, and calving date. Heifers F, H, and G were the youngest animals. The connection from F to any of the two others was (if present at all) very weak. Heifers G and H were associated in all constructed networks, which might be due to their age (four-day difference). The tendency that in mixed herds, cattle of the same breed show closer distances than expected by chance was supported by Stricklin, who investigated two mixed herds of Angus and Herford cows in a pasture [59]. Sato et al. observed more exchanges of allogrooming between cows of close birth dates [50].

The number of edges exceeded the number of nodes, which is known to be an important feature of animal social networks [24]. There were no striking hub nodes in the networks, which might be plausible since the animals form a herd and their demand for social contacts might be similar.

Animal H showed a small number of contacts, which might indicate that it prefers a larger social space; however, by considering all of its contacts, some of them appeared more often than expected by chance and might be desired ones by the animal itself.

## 5. Conclusions

In this study, we applied the PPMI followed by the APC procedure for the determination of animals’ associations and, thus, social network construction. Investigating the contact definition based on different distance thresholds, we concluded that the influence of the distance between animals on the network structure was marginal. We further examined the influence on animal activity (i.e., standing vs. lying) and found that the individual contact frequency, as well as the network structure differed between the two activities under study. This finding needs to be kept in mind for future contact studies and underlines the benefit of combined sensor usage. Regarding the animal associations, we found strong associations that were consistent throughout all constructed networks, and the corresponding heifers shared certain commonalities. Finally, we showed the robustness of the approach to missing data points and compared the approach to a state-of-the-art methodology (null models). The results of the proposed method were stable regarding a certain amount of missing data, which points out the potential for practical usage of the approach. Further, the findings of the null models were included in the results of the presented approach.

## Figures and Tables

**Table 1:** Overview of the heifers of the study. Each heifer received an capital letter for individual identification. All heifers were pregnant. The age and days before calving were determined by 24 September 2019 (study start) as the reference date. The column *Recordings* gives the number of GPS recordings per animal. The animal position was tracked with one-minute resolution. Observing the animals for 29 days, 1440×29 = 41,760 data points were recorded per animal without the loss of data points. The last column contains the mean number of satellites used for position estimation, as well as the mean dilution of precision (DOP).

Animal	Age (Months (Days))	Breed	Days b. Calving	Recordings	Mean Satellites (Mean DOP)
A	25 (+7)	Crossbreed	97	41,747	5.39 (1.53)
B	25 (+16)	Holstein Frisian	98	41,750	7.07 (1.51)
C	25 (+4)	Crossbreed	100	41,753	6.32 (1.52)
D	24 (+28)	Holstein Frisian	89	41,753	6.08 (1.49)
E	24 (+19)	Holstein Frisian	91	41,758	6.10 (1.49)
F	22 (+24)	Holstein Frisian	141	41,747	6.16 (1.50)
G	22 (+7)	Holstein Frisian	159	41,749	6.23 (1.50)
H	22 (+3)	Holstein Frisian	208	41,758	6.16 (1.50)

**Table 2:** Comparison between heifer pairs predicted by our ${PPMI}^{APC}$ approach with those showing a significant count or PMI values. The significant contact counts or PMI values were determined by creating 1000 samples based on shuffling the pairwise distance data. The contact counts or PMI values were significantly greater compared to those resulting from the random samples. An “x” indicates the presence of an edge.

Pair	${\mathrm{PPMI}}^{\mathrm{APC}}$	Sig. Contact Counts	Sig. PMI Values
A–C	x	x	x
B–E	x	x	x
D–E	x	x	x
A–F	x	x	x
C–F	x	x	x
E–G	x	x	x
D–G	x		x
D–H	x		x
G–H	x		x
A–B	x	x	
B–C	x	x	
B–F	x	x	
C–G	x	x	
E–H	x		
F–H	x		

**Figure A1:**
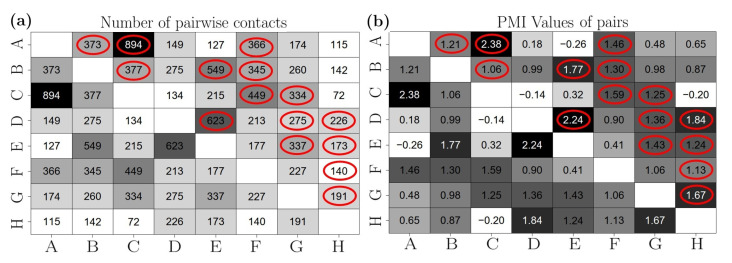
Matrix of the number of pairwise heifer contacts (**a**) and the corresponding PMI values (**b**). The red circles indicate that after APC procedure, the two entries formed an edge in the social network. Although the matrices are symmetric, we show all values to enable a better understanding of the functionality of PMI and the APC procedure. The background colors simplify the interpretation of the matrix; dark colors indicate high values and vice versa.

## Data Availability

The raw GPS data can be provided by the authors upon request. Please contact: sabrina.elsholz@uni-goettingen.de.

## Abbreviations

The following abbreviations are used in this manuscript:

APC	Average product correction
CEP	Circular error probability
DOP	Dilution of precision
GNSS	Global Navigation Satellite System
GPS	Global Positioning System
PMI	Pointwise mutual information
PPMI	Positive pointwise mutual information
RTK	Real-time kinematic
2DRMS	Twice-distance root mean square

## Appendix A

### Appendix A.1. Network Comparison of Different Distance Thresholds

**Table A1:** Comparison of the social network constructed for the different distance thresholds. An x indicates the presence of an edge (determined association between heifers) in the corresponding network.

Dyad	1 m	1.5 m	2.5 m	5 m
A–C	x	x	x	x
A–H				x
B–A	x	x	x	x
B–C	x	x	x	x
B–E	x	x	x	x
D–E	x	x	x	x
D–G	x	x	x	x
D–H	x	x	x	x
F–A	x	x	x	x
F–B	x	x	x	x
F–C	x	x	x	x
F–H	x			x
G–C	x	x	x	
G–E	x	x	x	x
H–E	x	x	x	x
H–G	x	x	x	x

### Appendix A.2. Understanding of PMI and the APC Procedure by Example

After counting the pairwise distances between heifers for each time point, we counted all contacts between heifers by using a distance threshold of 1 m and without making any constraints regarding the activity of the animals.

In the first step of the calculation, we used pointwise mutual information (PMI) in order to scale the associations with respect to the co-occurrence of heifers by pure chance. This step is shown in Figure A1, where the count values (a), as well as the corresponding PMI values (b) are presented. Most of the high count values had high PMI values. However, the order was changed in some cases, i.e., the pair C–G had a contact frequency of 334 and a PMI value of 1.25, whereas the pair D–G had 275 contacts and a PMI value of 1.35. Both pairs included G, and thus, the values related to B (Column B or Row B) in the column are not responsible for this flip of order. A look at the values belonging to C and D (contained in the corresponding rows) shows that C had three pairings having greater contacts as that with G. In turn, D only had one pair having more contacts and one having the same number of contacts as G. Thus, the interaction with G seemed to be much more important for D than for C, which is reflected by their PMI values.

After PMI calculation, we transformed the values to PPMI to avoid negative values and applied the APC procedure to eliminate the background arising from pure chance contacts. Simplified, the APC procedure calculates for each matrix entry the mean of the corresponding column and row and subtracts this from the matrix entry. It is obvious in Figure A1 that most of the small entries were eliminated by the APC procedure and the larger values remained (red circles). However, the pairs B–C and F–G both had a PMI value of 1.06, but only the pair B–C was included in the network. This was due to the fact that most of the PMI values of C were low, and thus, its average was smaller compared to the other IDs, leading to a positive value after average subtraction.
